# In Vivo High-Resolution 7 Tesla MRI Shows Early and Diffuse Cortical Alterations in CADASIL

**DOI:** 10.1371/journal.pone.0106311

**Published:** 2014-08-28

**Authors:** François De Guio, Sonia Reyes, Alexandre Vignaud, Marco Duering, Stefan Ropele, Edouard Duchesnay, Hugues Chabriat, Eric Jouvent

**Affiliations:** 1 Univ Paris Diderot, Sorbonne Paris Cité, UMR-S 1161 INSERM, Paris, France; 2 DHU NeuroVasc Sorbonne Paris Cité, Paris, France; 3 AP-HP, Lariboisière Hosp, Department of Neurology, Paris, France; 4 UNIRS, Neurospin, CEA, Gif-sur-Yvette, France; 5 Institute for Stroke and Dementia Research, Klinikum der Universität München, Ludwig-Maximilians-University, Munich, Germany; 6 Department of Neurology, Medical University of Graz, Graz, Austria; Nathan Kline Institute and New York University School of Medicine, United States of America

## Abstract

**Background and Purpose:**

Recent data suggest that early symptoms may be related to cortex alterations in CADASIL (Cerebral Autosomal-Dominant Arteriopathy with Subcortical Infarcts and Leukoencephalopathy), a monogenic model of cerebral small vessel disease (SVD). The aim of this study was to investigate cortical alterations using both high-resolution T2* acquisitions obtained with 7 Tesla MRI and structural T1 images with 3 Tesla MRI in CADASIL patients with no or only mild symptomatology (modified Rankin’s scale ≤1 and Mini Mental State Examination (MMSE) ≥24).

**Methods:**

Complete reconstructions of the cortex using 7 Tesla T2* acquisitions with 0.7 mm isotropic resolution were obtained in 11 patients (52.1±13.2 years, 36% male) and 24 controls (54.8±11.0 years, 42% male). Seven Tesla T2* within the cortex and cortical thickness and morphology obtained from 3 Tesla images were compared between CADASIL and control subjects using general linear models.

**Results:**

MMSE, brain volume, cortical thickness and global sulcal morphology did not differ between groups. By contrast, T2* measured by 7 Tesla MRI was significantly increased in frontal, parietal, occipital and cingulate cortices in patients after correction for multiple testing. These changes were not related to white matter lesions, lacunes or microhemorrhages in patients having no brain atrophy compared to controls.

**Conclusions:**

Seven Tesla MRI, by contrast to state of the art post-processing of 3 Tesla acquisitions, shows diffuse T2* alterations within the cortical mantle in CADASIL whose origin remains to be determined.

## Introduction

Cerebral autosomal-dominant arteriopathy with subcortical infarcts and leukoencephalopathy (CADASIL) is a rare hereditary cerebral small vessel disease (SVD) caused by mutations of the *NOTCH3* gene [1]. It is now widely recognized as a unique model for the study of more prevalent forms of SVD related to aging and hypertension [2]. The pathophysiology of SVD is thought to be mainly of subcortical origin, hence the term “subcortical ischemic vascular dementia” [3], but during the last decade the cerebral cortex was shown to be a key player in both sporadic forms of SVD and in CADASIL [4]–[8].

In a recent longitudinal study in CADASIL, processing speed slowing, but not global cognitive worsening nor increased disability was related to alteration of sulcal morphology, suggesting that early cognitive changes may be more specifically related to sulcal morphology than to other MRI markers [9]. The observed modifications of sulcal morphology could however be linked to underlying white matter structure changes [10] or to actual cortex involvement. The latter hypothesis could involve focal intra-cortical small-sized lesions [4], cortical demyelination [11] or increased iron content due to secondary degeneration, as reported previously in deep nuclei in CADASIL [12], all of which may translate in alterations of T2* measured using 7 Tesla MRI within the cortical mantle [13].

The aim of the present study was to investigate the cortex morphology using state of the art post-processing of 3 Tesla 3D T1 acquisitions together with its structure using high-resolution T2* 7 Tesla acquisitions in CADASIL patients at the early stage of the disease, with no or only mild symptomatology (modified Rankin’s scale ≤1 and MMSE ≥24), by comparison to age and sex matched controls.

## Methods

### Participants

The ethics committee of DRCD (Département de la Recherche Clinique et du Développement) of AP-HP (Assistance Publique-Hôpitaux de Paris) validated the protocol and all subjects gave their written consent for participating in the study. The present data originate from a prospective study of CADASIL patients with genetically confirmed diagnosis, without dementia (MMSE score ≥24) and without significant disability (modified Rankin’s scale ≤1) dedicated to the study of early cognitive symptoms in this disorder. Twenty-three patients and 29 age and sex matched controls were included in the study. Sixteen patients and 25 healthy controls had high quality 7 Tesla MRI covering the whole hemispheres and 3 Tesla MRI and were included in the present study. Clinical and demographic data were collected, including age, sex, smoking habits, alcohol intake, and body mass index. Laboratory evaluation (which included complete blood count, fasting glucose, high-density lipoprotein, low-density lipoprotein, and total cholesterol levels) was performed in all subjects. All participants underwent a comprehensive neuropsychological evaluation preceded by a clinical psychological interview. Global cognitive performances were assed using the Mini Mental State Evaluation (MMSE) [14]. Disability was assessed using the modified Rankin’s scale (mRS, from 0– no disability to 5– major disability), in which scores inferior or equal to one denote no disability [15]. An executive clock drawing task, CLOX [16] was used as a planning task, with both spontaneous and copy subtests. A verbal fluency task (phonological and semantic) [17] was administered and working memory was assessed using the working memory index from the Wechsler Memory Scale Third Edition (MEM-III) [18]. Specific computerized test were performed with various levels of executive demand but are not detailed here as they are not relevant to the present study. Presence of depression was defined following DSM-IV-TR criteria [19] and a depression rating scale, MADRS, was obtained for all subjects [20]. Subjects were considered apathetic if they had a Starkstein’s Apathy Scale score ≥14 and fulfilled the Starkstein Structured Clinical Interview for *Apathy* criteria [21].

### MRI protocol

Subjects underwent the same day both 3 Tesla and 7 Tesla MRI acquisitions at NeuroSpin (CEA, Gif-sur-Yvette, France). T_1_-weighted 3D images were acquired with a 3 Tesla Tim-Trio MRI scanner (Siemens Healthcare, Erlangen, Germany) equipped with a 12-channel head coil, using a standard sagittal MPRAGE sequence (in plane resolution: 1×1 mm^2^, slice thickness = 1.1 mm, TR = 2300 ms, TE = 2.98 ms, TI = 900 ms, FA = 9°, BW = 238 Hz/pixel, time of acquisition = 7′45 min). Ultra-high resolution MRI acquisitions were performed on a 7 Tesla MRI scanner (Siemens Healthcare, Erlangen, Germany) equipped with a 1Tx/8Rx head coil (Rapid Biomedical, Wurzburg, Germany). 2D axial gradient-echo sequences were acquired following AC-PC orientation, using seven or eight blocks depending on subject’ brain size. The choice of 2D blocks rather than whole brain 3D acquisition was made because of the impossibility for most subjects to remain free of movements during the necessary long acquisition periods. Each block comprised 20 slices with two different echo times using the following MR parameters: in plane resolution: 0.7×0.7 mm^2^, slice thickness = 0.7 mm, TR = 900 ms, TE_1_ = 13.7 ms, TE_2_ = 29.9 ms, FA = 65°, BW = 70 Hz/pixel, NA = 2, time of acquisition = 6 mn 46 s). Blocks were acquired parallel to each other with no overlap and were repeated, if possible, each time image was of poor quality. Total scan time by subject was about 1 hour on the 7 Tesla scanner. As FLAIR and T2* images were already obtained within 6 months on a 1.5 Tesla scanner (GE Healthcare, Milwaukee, Wisconsin) as part of an on-going prospective study in patients, theses sequences were used for the determination of white matter lesion volume and of number of microhemorrhages.

### Image processing

#### Quantification of subcortical MRI markers

Identification and quantification of MRI markers of SVD were performed according to recent standards [22]. White matter hyperintensity masks were automatically determined on all axial FLAIR slices from the base of the cerebellum to the vertex, using adaptative intensity thresholding [23]. Lacunes and dilated perivascular spaces were manually identified by experienced readers and lacune masks for each patient were automatically determined using simple connectedness algorithm with manual corrections when necessary [24]. All masks were manually validated in a second step by double reading, as previously reported [9]. Volumes were obtained by multiplying the number of voxels covered by the masks with their size depending on the sequence. The differentiation of lacunes from dilated perivascular spaces was based on current recommendations [22]. The number of cerebral microhemorrhages, defined as rounded foci smaller than 10 mm in diameter, hypointense on gradient-echo sequences, and distinct from vascular flow voids, leptomeningeal hemosiderosis, or non-hemorrhagic subcortical mineralization, was recorded.

#### Quantification of global and sulcal morphology

All images were processed using the BrainVisa suite (http://www.brainvisa.info). Brain parenchymal fraction (BPF) was defined as the ratio of brain volume to intracranial cavity determined on the 3D-T1 sequence, as previously reported in various disorders and in CADASIL [25]. BrainVisa was also used to reconstruct the cortical sulci [24]. Identification of sulci was made using a dedicated algorithm. For all subjects, results were carefully checked for both 2-dimensional segmentation masks and 3-dimensional reconstruction of sulcal shapes. Sulcal depth and sulcal width were obtained for 57 sulci on each hemisphere, and then averaged to determine for each subject mean sulcal width and mean sulcal depth.

#### Cortical thickness analysis

Three Tesla T_1_ images were processed using FreeSurfer version 5.1.0 (http://surfer.nmr.mgh.harvard.edu), which produced surface-based data for each subject [26]. FreeSurfer pipeline includes data resampling, standardized stereotaxic space normalization, intensity non-uniformity correction using N3, skull stripping, tessellation of the gray/white matter boundary, automated topology correction, and surface deformation to optimally delineate the gray/white matter and gray matter/cerebrospinal fluid boundaries [27], [28]. As previously reported, masks of white matter hyperintensities were obtained from FLAIR sequences and registered to 3D T1 images [9], [29]. Reconstructed datasets were always visually inspected for accuracy.

Cortical thickness maps were estimated by calculating the distance between the pial and white matter surface at each vertex from the 3 Tesla T1-weighted image [28]. For group analysis, each surface was spherically inflated and automatically registered to a canonical spherical template using cortical features. Thickness data from each subject were mapped to the average surface and smoothed using a Gaussian filter with a full-width half maximum of 10 mm before running general linear models (GLM). We contrasted CADASIL patients versus control subjects, covarying for age, gender and level of education. Thickness GLM results were corrected for multiple comparisons using a false discovery rate (FDR) of 0.05 [30].

#### Post-processing of 7 Tesla acquisitions

**Figure 1:**
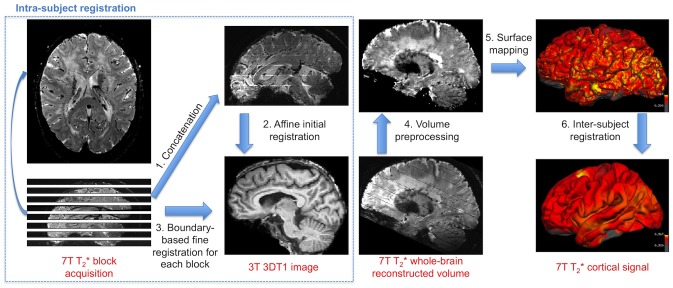
Image processing pipeline for surface-based intra-subject and inter-subject registration. **1.** Original 7 Tesla T2*-weighted blocks (axial image of a CADASIL patient is shown for example) are concatenated. **2.** Reconstructed volume is registered to the 3 Tesla T_1_-weighted image to produce an initial alignment. **3.** Each 7 Tesla block is then registered to the 3 Tesla image using the boundary-based registration algorithm initialized with the latter transformation. All blocks are combined to get a whole-brain 7 Tesla T2* reconstructed volume. **4.** Pre-processing include signal normalization by the first echo time image intensity, correction for intensity inhomogeneity and exclusion of null points corresponding to voids between blocks. **5.** 7 Tesla normalized T2* signal is mapped onto each individual’s cortical surface at the midline of the cortical ribbon to study cortical signal alterations. **6.** Each surface is smoothed and registered to a common template.

As high-resolution acquisitions at 7 Tesla were performed using multiple acquisitions of 2D slabs, a specific pipeline was set up in order to reconstruct the high-field whole brain image from those different blocks, as previously reported by another team in the context of multiple sclerosis [11]. The goal was to register the multiple 7 Tesla T2*-weighted blocks onto the 3 Tesla T1-weigthed anatomical scans (see Fig. 1).

*a) Intra-subject registration.* All 7 Tesla T2* blocks were first concatenated to create a rough whole brain reconstruction (Fig. 1). We then used the FSL FLIRT algorithm (http://www.fmrib.ox.ac.uk/fsl) to perform an initial affine registration between whole T2*-weighted volume at second echo time and 3D T_1_ volume which was over-sampled to an isotropic resolution of 0.7 mm. Next, boundary-based registration (BBR) was initialized with this latter transformation and ran separately for each block using the second echo time image for a better tissue contrast. BBR principle is to align the input image on the reference image by maximizing the intensity gradient across tissue boundaries [31]. BBR was particularly designed for aligning partial-brain images to whole-brain and for registering images with different contrasts. Quality of the block registration was visually checked systematically. If not satisfactory, manual adjustment was iteratively performed to change the initial conditions before BBR registration. Fine registered blocks were finally combined to create the reconstructed T2* volume. Data were dropped if registration still failed or if FreeSurfer pipeline was unable to reconstruct cortical surfaces.

*b) 7 Tesla volume pre-processing*. Potential null voxels corresponding to spaces between blocks were excluded from analysis. The reconstructed 7 Tesla T2* volume at second echo time was normalized by the first echo time volume and then corrected for non-uniformity intensity using N3 algorithm [27]. This was achieved to limit large spatial variations of signal due to B0 and B1 heterogeneities.

*c) 7 Tesla cortical signal analysis*. Signal at the midline of the cortical ribbon as defined by the internal and external surfaces computed by FreeSurfer was extracted from the normalized volume and mapped onto each subject’s cerebral surface. Each surface was then registered to the same average surface template of FreeSurfer with a 2D Gaussian smoothing (FWHM = 10 mm). Vertex-wise GLM was then run, contrasting CADASIL patients versus control subjects controlling for age, gender and level of education. Surface GLM results were corrected for multiple comparisons using a false discovery rate (FDR) of 0.05. Regions with significant differences were identified using an automatic parcellation of cortical gyri and sulci [32]. For each identified region, mean and standard deviation of 7 Tesla T2* signal and 3 Tesla cortical thickness were further calculated in the native space of each subject. Global T2* signal within the cortical mantle was also calculated for each subject in its native space. High quality data was obtained for 11 patients and 24 controls.

### Statistics

Statistical analyses reported in Table 1 were made using the R software (http://www.r-project.org/). For categorical variables, Chi2 tests were used. For numerical variables, Wilcox-tests were used given the small sample sizes.

**Table 1:** Characteristics of CADASIL patients and control subjects.

	CADASIL patients (N = 11)	Healthy controls (N = 24)	p-value
Age, mean ± sd, range	52.1±13.2, 32.1–70.6	54.8±11.0, 30.1–71.4	0.57
Male gender, n (%)	4/11 (36%)	10/24 (42%)	0.99
Right handed, n (%)	11/11 (100%)	24/24 (100%)	0.99
Level of education (years)	11.4±3.5	13.6±3.6	0.10
*Neuropsychological tests*			
MMSE, mean, median, range	28.5, 29, 24–30	29.0, 29, 26–30	0.45
Fluency letter p	24.1±9.8	25.2±7.9	0.82
Fluency category	22.1±6.1	22.3±4.4	0.75
Clox copy	14.8±0.4	14.8±0.4	0.84
Clox spontaneous	12.8±1.5	13.3±1.4	0.18
Working memory index	89.8±11.4	101.3±10.0	0.005
Apathy scale	9.6±3.1	6.8±2.9	0.03
Depression score	4.9±5.7	1.5±2.9	0.02
*Clinical data*			
SBP (mmHg)	122.3±10.5	128±14.8	0.35
DBP (mmHg)	75.6±5.8	77.4±14.5	0.90
Active smoking, n (%)	1/11 (9.1%)	4/24 (16.7%)	0.94
Glucose levels (g/l)	1.03±0.13	0.95±0.07	0.07
LDL-cholesterol (g/l)	1.09±0.29	1.27±0.29	0.18
HDL-cholesterol (g/l)	0.62±0.13	0.64±0.18	0.88
Triglycerides (g/l)	1.30±1.04	1.03±0.68	0.57
*Imaging markers*			
BPF	0.85±0.03	0.84±0.03	0.32
Mean sulcal width, mean ± sd (mm)	1.36±0.20	1.45±0.24	0.23
Mean sulcal depth, mean ± sd (mm)	13.41±0.84	13.76±0.47	0.22
White matter lesion volume, mean, median, range in mm^3^	65511.6, 47544, 7249.8–233797.3	No control had significant lesions	NA
Volume of lacunes, mean, median, range in mm^3^ (n = 5/11, 45%)^†^	423.5, 113.1, 14.0–1174.2	No control had lacunes	NA
Number of microhemorrhages, mean, median, range (n = 3/11, 27%)^†^	2.3, 1, 1–5	No control had microhemorrhage	NA

MMSE: Mini Mental State Examination, BPF: Brain Parenchymal Fraction, ^†^in patients with such lesions (number given in parentheses).

NA: not applicable.

## Results

Characteristics of both groups included in the analysis are detailed in Table 1. The two groups did not differ in terms of age, gender or MMSE (29 for median MMSE in both groups). BPF, mean sulcal width and mean sulcal depth did not either differ between groups. Patients differed relatively to controls in the working memory task and tended to have higher scores on apathy and depression scales.

### 7 Tesla cortical measures

**Figure 2:**
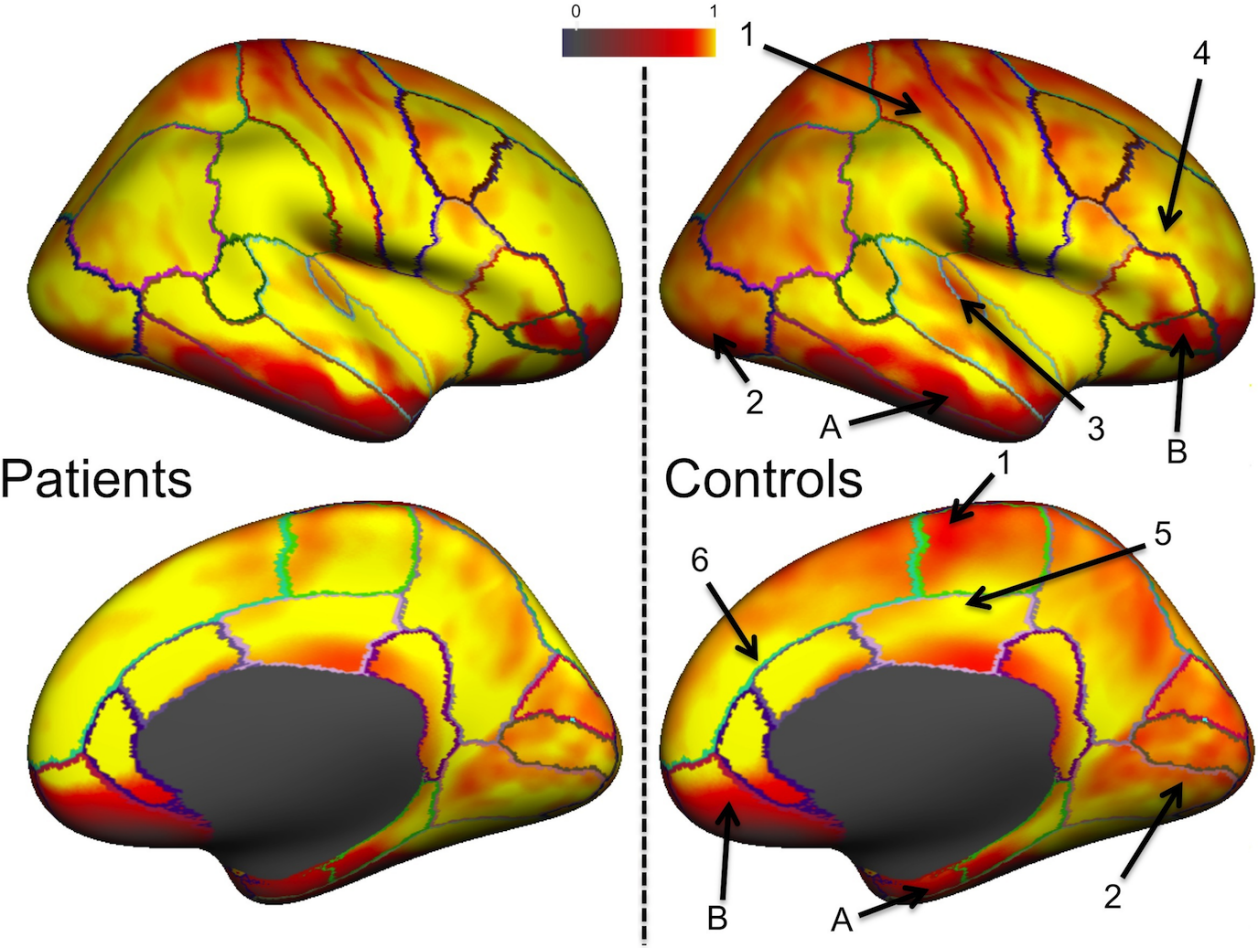
Normalized T2* signal in CADASIL and control subjects. Normalized T2* signal assessed at 7 Tesla across both groups is displayed on an inflated average surface for the right hemisphere. In both groups, hypointense signal is found around the central sulcus (**1**) due to high myelin content. As also previously reported, control subjects exhibit shorter T2* in visual (**2**) and auditory (**3**) areas and longer T2* in superior and middle frontal sulci (**4**) and in the posterior (**5**) and anterior (**6**) cingulate. Temporal (**A**) and frontal (**B**) signal loss due to susceptibility effects. CADASIL patients show an overall longer T2* compared to controls, leading to less variations between different cortical regions.

Mean normalized T2* signal for both groups is represented in Fig. 2. These cortical maps highlight regional differences across the cortex. While sensorimotor, visual and auditory areas exhibited lower values, higher values were found in the middle and superior frontal areas and in the cingulate cortex. Large hypointense inferior regions in frontal and temporal cortices were due to susceptibility effects. This pattern of T2* changes was found in both groups but differences between regions were visually smaller in CADASIL patients who showed a general increase in T2* in all cortical areas compared to control subjects. Mean T2* signal in the whole cortex was also found significantly higher in patients than in controls (0.73±0.07 vs 0.68±0.13, p = 0.01 after adjustment for age, gender and level of education).

**Figure 3:**
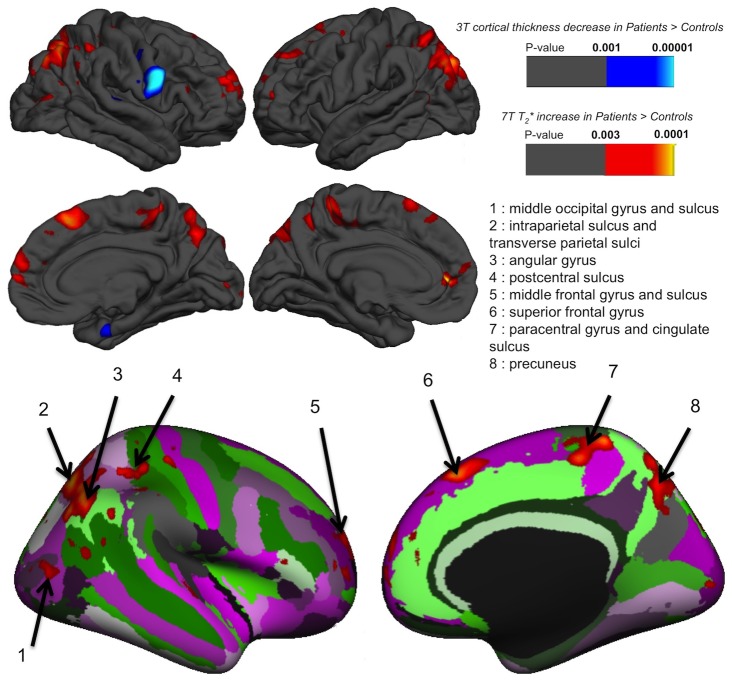
Surface-based GLM results: CADASIL vs Controls. GLM significance maps (p-value) overlaid on the average pial surface for both hemispheres on lateral (top row) and medial faces (middle row) for CADASIL patients (N = 11) versus control subjects (N = 24). 7 Tesla T2* and 3 Tesla cortical thickness differences were mapped in warm and cold colors, respectively. Results for T2* increase were also mapped for visualization on the right average inflated surface with cortical parcellation labeling (bottom row). There was no region in which CADASIL patients showed a significant reduced T2* compared to control subjects. By contrast, T2* was significantly higher in CADASIL patients in middle occipital gyrus and sulcus (**1**), intraparietal sulcus (**2**), angular gyrus (**3**), postcentral sulcus (**4**), middle frontal gyrus and sulcus (**5**), superior frontal gyrus (**6**), paracentral gyrus and cingulated sulcus (**7**) and precuneus (**8**). Cortical thickness was found reduced in patients only in the right precentral gyrus. Thresholds were set to FDR <0.05.

GLM results contrasting both groups showed several regions with a significant T2* increase in CADASIL patients compared to controls after adjustment for age, gender and level of education. As represented on the average pial surface, differences were largely symmetrical (Fig. 3). Table 2 reports significant local T2* changes between groups. There was no region where controls showed increased T2* compared to CADASIL patients. Higher T2* in patients with CADASIL were essentially located in parietal, frontal and occipital cortices. The left hemisphere showed more significant differences between groups. No significant correlation between T2* increase and BPF, white matter lesion volume, volume of lacunes or number of microhemorrhage were detected (data not shown). Comparing the 11 CADASIL patients with 11 age and sex matched controls yielded similar results with only reduced significance (see Figure S1). Mean cortical thickness in regions showing a difference in 7 Tesla T2* were not statistically different (Table 2).

**Table 2:** Cortical regions with a significant increase in 7 Tesla T2* in CADASIL patients compared to age and sex matched healthy subjects from the GLM analysis.

	Cortical area	p-value^1^	Cluster size^2^ (mm^2^)	Talairach coordinates			ROI T2* signal				ROI cortical thickness (mm)				
				x	y	z	CADASIL		Controls		CADASIL		Controls		p-value^3^
							mean	sd	mean	sd	mean	sd	mean	sd	
*Left*															
	Anterior cingulate gyrus and sulcus	7.1×10^−6^	184.54	−8.2	44.9	1.8	0.85	0.08	0.77	0.14	2.42	0.23	2.40	0.21	0.54
	Paracentral lobule and sulcus	9.5×10^−6^	321.24	−8.8	−39	59.1	0.78	0.08	0.66	0.14	2.30	0.16	2.41	0.19	0.07
	Fronto-marginal gyrus and sulcus	2.8×10^−5^	58.24	−23.6	51.6	−1.9	0.84	0.08	0.71	0.15	2.46	0.23	2.40	0.23	0.26
	Superior occipital gyrus	3.1×10^−5^	597.53	−22.6	−78.7	38	0.81	0.08	0.70	0.14	2.39	0.22	2.41	0.18	0.99
	Angular gyrus	6.2×10^−5^	681.73	−40.8	−73	36	0.83	0.08	0.73	0.15	2.38	0.17	2.42	0.12	0.44
	Superior parietal lobule	1.3×10^−4^	374.21	−14.3	−74.6	46	0.79	0.08	0.69	0.13	2.41	0.17	2.40	0.15	0.73
	Superior parietal lobule	2.5×10^−4^	320.59	−22.1	−60.5	53.8	0.79	0.07	0.69	0.14	2.35	0.25	2.38	0.21	0.86
	Superior frontal gyrus	3.8×10^−4^	102.05	−4.7	21.2	58.6	0.69	0.08	0.56	0.14	2.38	0.15	2.42	0.13	0.43
	Superior frontal gyrus	4.5×10^−4^	106.22	−17.6	46.5	35.6	0.76	0.07	0.64	0.12	2.35	0.23	2.42	0.16	0.48
*Right*															
	Intraparietal sulcus	4.3×10^−5^	3112.85	21.3	−60.4	44.9	0.80	0.07	0.71	0.14	2.45	0.20	2.40	0.16	0.43
	Superior frontal gyrus	5.9×10^−5^	488.89	4.4	27.2	46.2	0.69	0.08	0.57	0.14	2.50	0.34	2.50	0.32	0.92
	Paracentral lobule and sulcus	7.8×10^−5^	412.82	11.6	−36.9	60.2	0.74	0.09	0.62	0.14	2.41	0.18	2.39	0.22	0.62
	Superior frontal gyrus	3.0×10^−4^	1165.26	17.5	55.5	20.5	0.80	0.06	0.70	0.14	2.40	0.19	2.47	0.19	0.27
	Intraparietal sulcus	3.2×10^−4^	286.68	36.5	−42.4	48.6	0.78	0.06	0.70	0.13	2.39	0.25	2.40	0.23	0.88
	Postcentral gyrus	3.3×10^−4^	139.72	39.5	−22.5	57.2	0.74	0.08	0.64	0.15	2.44	0.26	2.39	0.16	0.35
	Superior occipital gyrus	5.8×10^−4^	233.97	11	−84.7	34.9	0.77	0.07	0.66	0.14	2.39	0.30	2.34	0.23	0.37
	Angular gyrus	6.3×10^−4^	109.8	54.8	−51.8	32.1	0.83	0.07	0.75	0.15	2.41	0.17	2.41	0.21	0.68
	Middle occipital gyrus	6.7×10^−4^	198.66	45.5	−76.8	12.2	0.78	0.07	0.70	0.14	2.41	0.31	2.33	0.26	0.36
	Middle frontal sulcus	7.0×10^−4^	73.3	29.7	48	3.7	0.80	0.09	0.70	0.15	2.39	0.29	2.40	0.25	0.82
	Middle frontal gyrus	9.4×10^−4^	52.24	39.1	52.8	8.6	0.79	0.05	0.72	0.15	2.50	0.18	2.40	0.20	0.08
	Postcentral sulcus	1.2×10^−3^	58.37	43.5	−33.5	42	0.80	0.08	0.74	0.14	2.31	0.29	2.30	0.28	0.83
	Transverse temporal sulcus	1.3×10^−3^	70.34	57.4	−11.8	4.2	0.79	0.07	0.67	0.16	2.25	0.35	2.36	0.35	0.28
*Global*							0.73	0.07	0.68	0.13	2.59	0.14	2.57	0.09	0.49

Cortical thickness measurements in these regions of interest (ROI) are also reported.

1 max uncorrected p-value in the cluster, p_right_ >0.003, p_left_ >0.001 (False discovery rate = 0.05);

2 cluster area >50 mm^2^;

3 adjusted for age, gender and level of education (p-value obtained at the cluster level).

### Cortical thickness

GLM results contrasting both groups showed no significant differences between CADASIL patients compared to controls after adjustment for age, gender and level of education, except a single area showing reduced thickness in patients in the precentral gyrus of the right hemisphere (Fig. 3). Mean thickness over the whole cortex was not different between controls and CADASIL patients (2.59±0.14 mm vs 2.57±0.09 mm, p = 0.49 after adjustment for age, gender and level of education).

### Relationships between T2* and clinical status

None of the neuropsychological variable was found significantly associated with T2* cortical signal using FDR threshold. Some marginal associations were observed using a more permissive threshold (p<0.05) as represented in Figure S2.

## Discussion

In the present study, we observed a strong and diffuse T2* increase within the cortical mantle in CADASIL patients with no or only mild symptomatology by comparison to age and sex matched controls. By contrast, conventional state-of-the art post-processing of high-resolution 3D T1 images showed only minor, if any, group differences. The increased T2* was significant in various frontal and parietal regions most often following a symmetrical pattern on both hemispheres. These T2* alterations were not related to cortical thinning.

The observed increase of T2* at ultra-high field in patients could have several explanations. Indeed, T2* signal mostly relies on proton density and spin phase coherence between protons. When proton density or spin phase coherence increase, then the T2* signal is increased. The presence of intracortical infarcts, as previously depicted pathologically in a CADASIL patient [4], could lead to an increased T2* through an increase of proton density because of the presence of intracortical fluid-filled cavities, but this hypothesis has been ruled out by visual inspection although we cannot formally exclude the presence of numerous cortical microinfarcts not visible on MRI. However, this appears very unlikely given that the patients were all at the early stage of the disease.

The increase of T2* could also be secondary to a reduction of the loss of spin phase coherence between protons. In the cortex, both myelin and iron contribute to the T2* relaxation, with a predominant role of iron [33], [34]. Decreased iron concentration or demyelination could be involved in the T2* increase according to MR tissue contrast modeling from both histology and post-mortem MRI scanning [34]. By contrast, secondary degeneration related to the accumulation of lacunar lesions in subcortical areas, as reported on conventional imaging recently [10], is unlikely explaining our results as it would rather lead to a decrease of the T2* signal because of an increased iron concentration as confirmed pathologically in basal ganglia [12].

As deoxygenated red blood cells are paramagnetic and induce loss of spin phase coherence, another possible origin of these T2* increase within the cortex might be the variations in venous vascular density and/or blood oxygenation [35]. A reduction of vascular density, in line with observations reported in a mouse model of CADASIL would imply less spin dephasing and thus increased T2* [36]. Capillary loss has also been observed pathologically in the cortex of patients with extensive white-matter hyperintensities [37], and we have recently reported using 7T MRI an increase of T2* within the white matter in CADASIL patients having less visible veins in the centrum semiovale [38].

Otherwise, myelin-associated iron may also be a major source of intracortical magnetic susceptibility-based contrast [33]. In multiple sclerosis, T2* increase observed at 7 Tesla have been associated with general demyelination [11]. Edema may also be involved in the observed T2* increase. We have reported that brain volume was increased in CADASIL patients with high loads of white matter hyperintensities [39] and a recent study in a mouse model of CADASIL have shown an intramyelinic edema as soon as the early stage of the disease, without blood-brain-barrier involvement [40]. Other potential sources of increased T2* at ultra-high field include inflammation and gliosis [41], but to our knowledge, they have not been reported to be diffuse in the cortex of CADASIL patients.

In line with previous data, which showed alterations of the cortex morphology that parallel the clinical worsening in CADASIL [9], our findings emphasize the role of the cerebral cortex in CADASIL and more generally in SVD. Our results suggest that cortex alterations may appear early during the course of the disease, when patients still have no or only mild symptomatology and before global brain atrophy or cortical atrophy could be demonstrated using conventional imaging. By contrast to previous works, we did not observe cortical thinning in patients compared to controls [10], but in the present study, patients were less severe. Moreover, by comparison to previous studies in CADASIL, patients had lower loads of lacunar lesions in the present study, which may explain why cortical thinning could not be detected. The single area with decreased cortical thickness in right precentral gyrus should be interpreted cautiously given the multiple testing approaches in vertex wise analyses and this result may be due to chance alone.

In this study, we did not observe any significant relationship between clinical variables and the increase of T2* signal in the cerebral cortex when taking into account multiple testing. When using a less conservative threshold, only minor associations were detected (Figure S2). Given the clinical differences between patients and controls, which were very small, it would have been surprising to find strong correlations with the clinical status. Further studies will be needed to determine whether these alterations are clinically meaningful.

The main limitation of our study is the small number of patients as a major source of data loss was the incapacity to undergo long lasting acquisition (more than one hour in the 7 Tesla scanner). Importantly, results were left unchanged when the 11 patients were compared to 11 age and sex matched controls, with only a slight decrease in the level of significance (see Figure S1). Thus, our results could unlikely be explained by chance or selection biases. Moreover, the post-processing of images used in the present study is complex, involving multiple steps for both 3 Tesla and 7 Tesla acquisitions, which could have induced various biases and errors. Natives images were thus carefully checked and T2* measured at multiple positions, confirming the clear tendency to T2* increase in patients. Moreover, as patients did not show measurable global or cortical atrophy, these potential sources of systematic errors can be realistically excluded.

Our study has also several strengths. High-field 7 Tesla T2* acquisition combined to an isotropic 0.7 mm^3^ resolution produced unique data to assess cortical T2* variations with reduced partial volume effects. The block acquisition protocol enabled to rescan individual blocks during the imaging session if subjects moved. The surface-based intra- and inter-subject registration strategy made possible to overcome individual anatomical variability to probe global cortical signal changes.

In summary, the use of 7 Tesla MRI high-resolution acquisitions allowed demonstrating strong and early involvement of the cortical T2* in CADASIL, undetected using state of the art post-processing analyses of conventional 3 Tesla MRI images. Further studies are needed to understand the underlying mechanisms of these alterations and their clinical consequences as well as to determine whether our findings can extend to more frequent forms of small vessel disease of the brain.

## Supporting Information

Figure S1 Effect of smaller control sample on surface-based GLM results. GLM significance maps (p-value) overlaid on the average left pial surface for the main analysis (right, 11 patients vs. 24 controls) and for a matched number of controls (left, 11 patients vs. 11 controls).
(TIFF)

Figure S2 Association between cortical T2* and other variables. GLM significance maps (p-value) overlaid on the average inner cortical surface for both hemisphere after adjustment for age, sex and gender for several variables: apathy, depression, working memory, MMSE (Mini Mental State Examination), Verbal fluency and WMH (white matter hyperintensities) volume.
(TIFF)
