# Supplementary figures and images for: In Vivo High-Resolution 7 Tesla MRI Shows Early and Diffuse Cortical Alterations in CADASIL
Source: PLoS One. 2014 Aug 28;9(8):e106311. doi: 10.1371/journal.pone.0106311 (PMC4148432; doi:10.1371/journal.pone.0106311)

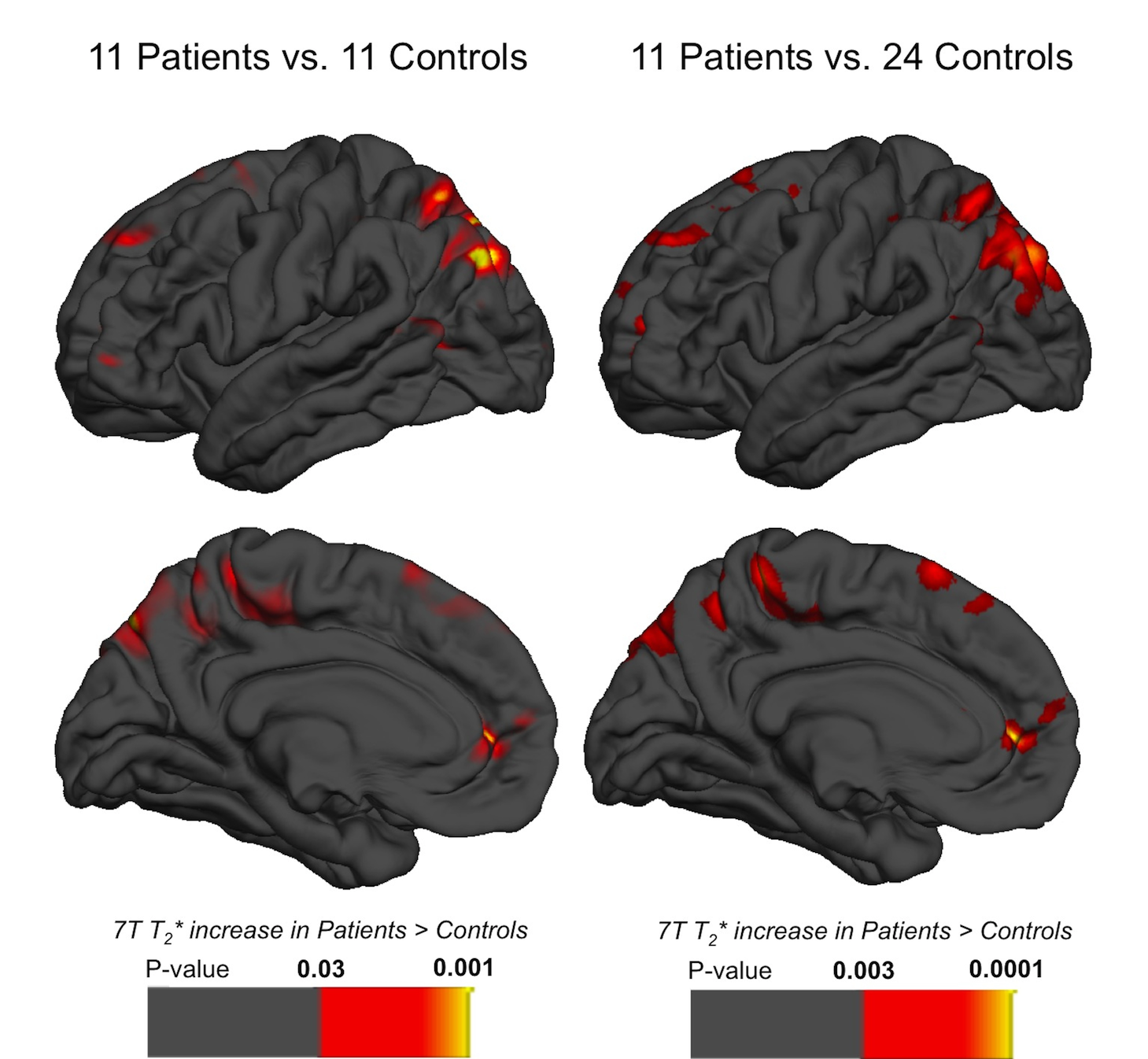

Supplement: Figure S1 — Effect of smaller control sample on surface-based GLM results. GLM significance maps (p-value) overlaid on the average left pial surface for the main analysis (right, 11 patients vs. 24 controls) and for a matched number of controls (left, 11 patients vs. 11 controls). (TIFF) [file pone.0106311.s001.tiff]

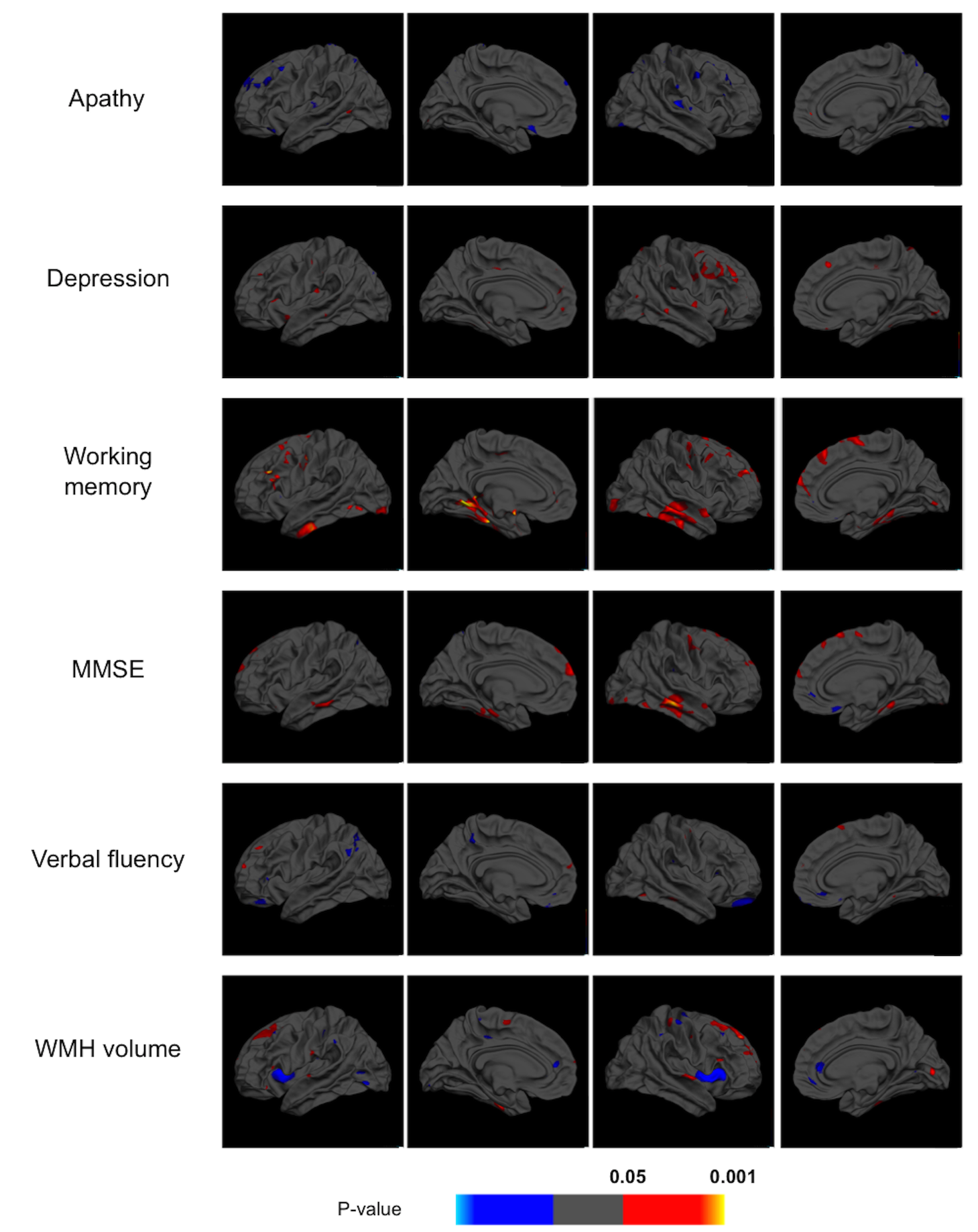

Supplement: Figure S2 — Association between cortical T2* and other variables. GLM significance maps (p-value) overlaid on the average inner cortical surface for both hemisphere after adjustment for age, sex and gender for several variables: apathy, depression, working memory, MMSE (Mini Mental State Examination), Verbal fluency and WMH (white matter hyperintensities) volume. (TIFF) [file pone.0106311.s002.tiff]
